# Membrane binding, internalization, and sorting of alpha-synuclein in the cell

**DOI:** 10.1186/s40478-018-0578-1

**Published:** 2018-08-14

**Authors:** Caterina Masaracchia, Marilena Hnida, Ellen Gerhardt, Tomás Lopes da Fonseca, Anna Villar-Pique, Tiago Branco, Markus A. Stahlberg, Camin Dean, Claudio O. Fernández, Ira Milosevic, Tiago F. Outeiro

**Affiliations:** 1 Department of Experimental Neurodegeneration, Center for Biostructural Imaging of Neurodegeneration, Center for Nanoscale Microscopy and Molecular Physiology of the Brain, University Medical Center Goettingen, 37073 Göttingen, Germany; 2 Max Planck Laboratory for Structural Biology, Chemistry and Molecular Biophysics of Rosario (MPLbioR, UNR-MPIbpC) and Instituto de Investigaciones para el Descubrimiento de Fármacos de Rosario (IIDEFAR, UNR-CONICET), Universidad Nacional de Rosario, Ocampo y Esmeralda, S2002LRK Rosario, Argentina; 3 European Neuroscience Institute, University Medical Center Göttingen, Göttingen, Germany; 4 Trans-synaptic Signaling Group, European Neuroscience Institute, Grisebachstrasse 5, 37077 Goettingen, Germany; 5 Max Planck Institute for Experimental Medicine, Göttingen, Germany; 6 Institute of Neuroscience, The Medical School, Newcastle University, Framlington Place, Newcastle Upon Tyne, NE2 4HH UK

**Keywords:** Alpha-synuclein, Parkinson’s disease, Uptake, Spreading, Rab proteins

## Abstract

Alpha-synuclein (aSyn) plays a crucial role in Parkinson’s disease (PD) and other synucleinopathies, since it misfolds and accumulates in typical proteinaceous inclusions. While the function of aSyn is thought to be related to vesicle binding and trafficking, the precise molecular mechanisms linking aSyn with synucleinopathies are still obscure. aSyn can spread in a prion-like manner between interconnected neurons, contributing to the propagation of the pathology and to the progressive nature of synucleinopathies. Here, we investigated the interaction of aSyn with membranes and trafficking machinery pathways using cellular models of PD that are amenable to detailed molecular analyses. We found that different species of aSyn can enter cells and form high molecular weight species, and that membrane binding properties are important for the internalization of aSyn. Once internalized, aSyn accumulates in intracellular inclusions. Interestingly, we found that internalization is blocked in the presence of dynamin inhibitors (blocked membrane scission), suggesting the involvement of the endocytic pathway in the internalization of aSyn. By screening a pool of small Rab-GTPase proteins (Rabs) which regulate membrane trafficking, we found that internalized aSyn partially colocalized with Rab5A and Rab7. Initially, aSyn accumulated in Rab4A-labelled vesicles and, at later stages, it reached the autophagy-lysosomal pathway (ALP) where it gets degraded. In total, our study emphasizes the importance of membrane binding, not only as part of the normal function but also as an important step in the internalization and subsequent accumulation of aSyn. Importantly, we identified a fundamental role for Rab proteins in the modulation of aSyn processing, clearance and spreading, suggesting that targeting Rab proteins may hold important therapeutic value in PD and other synucleinopathies.

**Electronic supplementary material:**

The online version of this article (10.1186/s40478-018-0578-1) contains supplementary material, which is available to authorized users.

## Introduction

The aggregation and accumulation of proteins in the brain is a common feature among several neurodegenerative disorders such as Parkinson’s disease (PD) and dementia with Lewy bodies (DLB) [24]. These diseases are part of a group known as synucleinopathies, characterized by the accumulation of proteinaceous inclusions enriched in alpha-synuclein (aSyn) [55, 56, 59, 60, 64], an abundant protein in the brain that is found in presynaptic terminals and also in other subcellular compartments. The precise physiological function of aSyn remains elusive, but it is thought to be involved in synaptic vesicle trafficking and biology [47]. aSyn can be divided into three distinct regions based on the amino acid composition: the N-terminal region (residues 1–60) adopts amphipathic α-helical structure when associated with membranes ([13, 15]; the central region (residues 61–95) is highly hydrophobic and essential for aggregation ([23]; and the C-terminal region (residues 96–140) is enriched in acidic residues and is involved in several protein-protein interactions [26], conferring to the protein a chaperone-like function [31, 43, 54]. aSyn is an intrinsically disordered protein (IDP), characterized by the lack of defined secondary structure under physiological conditions. However, aSyn adopts helical structure in the first 100 residues upon interaction with membranes [21, 28, 51].

The precise physiological form of aSyn is still a matter of debate. Initially, the protein was thought to be monomeric, existing in a balanced equilibrium between a cytosolic, free state, and a state bound to the plasma membrane and vesicles. Recently, multimeric forms of aSyn, mainly tetrameric, have been isolated from various cell types [3, 9, 65], initiating a debate about its natural state.

Several mutations in the *SNCA* gene have been identified in familial forms of PD (A53T [45], A30P [32], E46K [67], H50Q [2], G51D [35] and A53E [44]). In addition, overexpression of wild-type aSyn (aSyn WT) due to duplication [16] or triplication [49] of the *SNCA* gene are also associated with autosomal dominant forms of PD.

Intense efforts have focused on the study of the molecular mechanisms underlying aSyn misfolding and aggregation. Recently, cell-to-cell spreading of aSyn has become an attractive model to explain the progressive nature of these diseases and the typical patterns of pathology deposition in neuroanatomically connected regions of the diseased brain. Multiple studies demonstrated that aSyn oligomers and pre-formed fibrils (PFFs) enter cultured cells and accumulate in the cytoplasm [37, 38, 63]. However, it is still unclear how aSyn enters cells and where aggregation starts. The hypothesis that aSyn multimerizes upon interacting with lipid membranes [9] raised the question of whether α-helical aSyn multimers directly transition into β-strand-rich cytotoxic forms, or whether it is the unstructured, monomeric form that transitions to aggregates inside cells, during the processing and compartmentalization in different organelles and the interaction with effector proteins.

We have previously shown that small Ras-like GTPases (Rabs) proteins, key mediators of the membrane trafficking and vesicle recycling, can also modulate aSyn oligomerization and aggregation [5, 17, 25]. Rabs act as molecular “switches” that alternate between two conformational states: the GTP-bound ‘on’ form, and the GDP-bound ‘off’ form [57]. Notably, mutations in RAB genes (e.g. *RAB7L1 and RAB39B*) and in their regulators or effectors have been implicated in several neurological and neurodevelopmental disorders, suggesting that impairment in the function of these proteins might be linked to familial forms of PD [36, 48, 66].

Here, we investigated the internalization of aSyn and the role of Rab proteins in mediating this process. We found that membrane interactions by aSyn enable internalization and that Rab proteins mediate the intracellular distribution of the protein. In total, our study provides novel insight into the molecular mechanisms associated with aSyn internalization and aggregation, paving the way for future intervention strategies aimed at interfering with the spreading of aSyn pathology.

## Materials and methods

### aSyn purification

aSyn WT, aSyn A30P and aSyn A11P/V70P were obtained by transforming *E.coli* BL21-DE3 competent cells with plasmids encoding corresponding cDNA sequences (pET21-aSyn, pET21-A30P, pET21-A11P/V70P).

Purification was performed as previously reported [26] with minor modifications. Briefly, BL21-DE3 cells were grown in LB medium in the presence of ampicillin (100 μg/ml). Protein expression was induced with 1 mM IPTG for 4 h at 37 °C. Afterwards, cultures were harvested and the cell pellet was resuspended in Lysis Buffer (50 mM Tris HCL, 150 mM NaCl, 1 mM EDTA and Inhibitor Protease cocktail) at pH 8.0. Cells were recovered, sonicated on ice, boiled for 20 min at 95 °C, and cell debris were discarded by centrifugation. Subsequent precipitation first with streptomycin sulphate (10 mg/ml) and later with ammonium sulphate (361 mg/ml) was used to obtain aSyn-enriched precipitate.

Anion exchange high-performance liquid-chromatography (AEC) was carried out on an Äkta-HPLC Purifier (GE Healthcare). The pellet was resuspended then in 25 mM Tris-HCl (pH 7.7), and loaded onto a Mono Q column or bounded to a Hi-Trap column (GE Healthcare). The monomeric proteins were eluted at ∼300 mM NaCl with a linear salt gradient of elution buffer from 0 mM to 1 M NaCl. The pure proteins (judged by PAGE) were dialyzed overnight against the appropriate buffer and further size exclusion chromatography (SEC) purification step using a Superdex 75 column (GE Healthcare) was performed.

Protein concentration was estimated from the absorbance at 274 nm using an extinction coefficient of 5600 M^− 1^ cm^− 1^. The protein stocks were frozen in single aliquots at − 80 °C.

### Fibril formation

Three aliquots of 300 μL of aSyn WT were prepared from the protein stocks, and diluted in phosphate saline buffer (PBS) to reach a final concentration of 60 μM. Samples were incubated in an Eppendorf Thermomixer Comfort (Eppendorf, USA) with 0.02% sodium azide at 600 rpm and 37 °C.

The transition of aSyn from initial soluble monomeric form to aggregated state was determined by measuring light scattering in a Jasco FP-8200 spectrofluorometer (Jasco Inc., MD, USA) with an excitation wavelength of 330 nm and emission range from 320 to 340 nm at 25 °C. Solutions without protein were used as negative controls. All experiments were carried out in triplicates.

### Cell line cultures and treatments with aSyn

Human neuroglioma H4 cells were maintained at 37 °C and 5% CO_2_ environment, in Opti-MEM medium (PAN, Germany) supplemented with 10% fetal calf serum (ThermoFisher) and 1% penicillin-streptomycin (ThermoFisher).

Cells were seeded in different well-plate formats, one day prior to transfection, at a density of 8.5 to 1*10^5^ cells/ml. The day after, cells were treated with different concentrations of aSyn monomers or aSyn fibrils for 24 h. At the end of the treatment, cells were extensively washed with PBS and then briefly treated with trypsin in order to remove the residual proteins still outside of the cells or bound to the dish (for a maximum time of 30 s), incubated again with medium (in order to stop the trypsinization) and then washed one last time with PBS.

Transfections were performed with calcium phosphate following the procedure from www.flemingtonlab.com. Shortly, 3 h prior to transfection, fresh cell medium was added to the cells. DNA was diluted in 1× HBS buffer with 25 mM 4-(2-hydroxyethyl)-1-piperazineethanesulfonic acid, 140 mM NaCl, 5 mM KCl, 0.75 mM Na_2_HPO_4_ ·2H_2_O, 6 mM Dextrose, pH 7.1. After mixing, 2.5 M CaCl_2_ was added dropwise and vigorously mixed. Followed 20 min of incubation, the mixture was added dropwise to the cells. In the next morning cells were fed with fresh medium.

### Immunoblot analysis

Cells were solubilised with RIPA buffer (50 mM Tris pH 8.0, 150 mM NaCl, 0.1% Sodium-Dodecyl-Sulphate (SDS), 1% Nonidet P40, 0.5% Sodium-Deoxycholate, protease inhibitors) and protein quantification was done using the Bradford assay (BioRad). All samples were measured in triplicate.

Cell lysates were separated by SDS-PAGE under reducing conditions in 12% separating gels with 7% stacking gels. After electrophoresis, proteins were transferred onto 0.45 μm nitrocellulose membranes for 20 min per membrane at constant 25 mA in a semi-dry transfer chamber Trans-Blot® Turbo™ Transfer Solution from Bio-Rad (Bio-Rad Laboratories, Inc., Hercules, CA, USA). Free binding sites were blocked with 10% (*w*/*v*) skim milk dissolved in TBS-T (50 mM Tris (hydroxymethyl)-aminomethane (TRIS) supplemented with 0.05% (*v*/v) Tween-20) for 1 h at Room Temperature (RT).

For detection, the primary antibodies were dissolved in TBS and incubated over night at 4 °C. Detection of proteins on immunoblots was performed to detect aSyn, (aSyn C-20, 1:1000, Santa-Cruz Biotechnology) β-actin (Actin beta, 1:10.000, Sigma Aldrich), tubulin (tubulin β-III, 1:10.000, Santa-Cruz Biotechnology), transferrin receptor (Tfr Receptor, 1:1000, Invitrogen-Life Technologies) and Rab GTPases fused to GFP (GFP Ab, 1:1000, Roche). After washing with TBS-T, secondary HRP-conjugated antibodies (GE Healthcare) were diluted 10,000-fold in TBS-T and incubated with the membrane for 2 h at RT.

Membranes were visualized using Fusion Fx (Vilber Lourmat, Marne-la-Vallée, France) with Immobilon Western Chemiluminescent HRP Substrate (Merck Millipore, Billerica, MA, USA).

### Dot blot analysis

All HPLC samples were boiled for 10 min at 95 °C at 650 rpm and then spun down briefly at 10,000 g and 4 °C. Samples were loaded fully onto a 96 well custom-manufactured Dot Blot machine. A vacuum pump was used to suck sample through a 0.2 μm pore size Protean nitrocellulose membrane (Schleicher & Schuell Bioscience GmbH, Dassel, Germany). The membrane was subsequently blocked with 5% skim milk in TBS (to prevent unspecific staining) for 1 h.

Membranes were incubated with primary antibody (aSyn BD transduction, 1:2000; BD Biosciences) diluted in 1% skim milk in TBS, or 5% bovine serum albumin (BSA) in TBS, overnight at 4 °C. At the end of the incubation, membranes were washed three times with TBS-T for 10 min.

Subsequently, membranes were incubated with HRP-conjugated secondary antibody (GE Healthcare) diluted 1:10,000 in TBS. Afterwards, membranes were visualized using Fusion Fx (Vilber Lourmat, Marne-la-Vallée, France) with Immobilon Western Chemiluminescent HRP Substrate (Merck Millipore, Billerica, MA, USA).

### Triton X-100 fractionation assay

Cells were plated and treated as described above. At the end of the treatment, cells were lysed in Lysis Buffer I (25 mM Tris pH 7.5, 150 mM NaCl, 1 mM EDTA and cocktail of protease inhibitors) and centrifuged at 100.000 g for 30 min at 4 °C. Supernatants were collected (soluble fraction) and the pellets (insoluble fraction) were washed with cold PBS and transferred to new tubes. Samples were centrifuged once again 14.000 rpm for 10 min at 4 °C and the resulting pellet, corresponding to the insoluble fraction, was subsequently resuspended in Lysis Buffer II (75 mM Tris, pH 6.8, 3% SDS, 15% Glycerin, 3.75 mM EDTA pH 7.4 and a cocktail of protease inhibitors). Finally, samples were sonicated (10 pulse/second) and immunoblotting analysis were performed as described above.

### Membrane biotinylation assay

The day before the experiment, cells were plated in 100 mm Petri dishes at a density of 4*10^6^ cells, and grown until 60–70% confluence. Thereafter, cells were treated with 1 μM of aSyn recombinant monomers or fibrils of different aSyn variants (aSyn WT, aSyn A30P or aSyn A11P/V70P) for 24 h.

At the end of the treatment, cells were treated as previously described [20, 46]. Briefly, cells were rinsed 3 times in ice-cold PBS and further incubated in PBS containing 1.5 mg/ml of EZ-Link Sulfo-NHS-SS-Biotin (ThermoFisher) with gentle rocking, for 30 min, at 4 °C. The non-bound biotin was removed by incubating cells with 100 mM solution of Glycine for 15 min at 4 °C.

To remove the excess of glycine, cells were briefly washed with PBS and thereafter cell lysate was prepared in PBS containing Protease Inhibitor α-complete (La Roche, Basel, Switzerland), 0.1% SDS and 1% Triton X-100. The lysates were sonicated for 30 s and centrifuged for 5 min at 17000 × g. The supernatant was further incubated with 100 μL of NeutrAvidin Agarose Resin (ThermoFisher) for 2 h, in a rotatory shaker with gentle agitation, at 4 °C. After the incubation with the resin, the supernatant (corresponding to the Cytoplasmic cell lysate fraction) was collected, and a Bradford assay was performed to evaluate the amount of total protein concentration in each of the samples.

Biotinylated proteins were then washed 3 times with PBS and then eluted with 2× Laemmli Buffer by boiling the samples at 95 °C for 5 min.

Samples were then processed by western blotting. Transferrin receptor was used as a positive control of the biotinylated fraction, whereas tubulin was used as a positive control for the cytoplasmic cell lysate fraction.

### Immunocytochemistry (ICC)

For ICC analysis, cells were plated in multi-well plates with different formats, previously coated with coverslips. 24 or 48 h after transfection, H4 cells were washed with PBS and fixed with 4% paraformaldehyde (PFA) for 10 min at RT, followed by a permeabilisation step with 0.5% Triton X-100 (Sigma Aldrich, St. Louis, MO, USA) for 10 min at RT.

After blocking in 10% normal goat serum (PAA, Cölbe, Germany)/DPBS for 1 h, cells were incubated with primary antibody. Primary antibodies used were: rabbit anti-aSyn (Human aSyn (C-20): sc-7011-R, 1:1000, Santa Cruz Biotechnology, Dallas, USA) or mouse anti-aSyn (Human aSyn, 610,787, 1:2000, BD Transduction), for 3 h or overnight and secondary antibody (Alexa Fluor 555 donkey anti-mouse IgG and/or Alexa Fluor 555 goat anti rabbit IgG, (Life Technologies- Invitrogen, Carlsbad, CA, USA) for 2 h at RT. In some of the experiments cells were incubated with Phalloidin (Phalloidin 488, A12379, Phalloidin 594, A12381, 1:50 in PBS, ThermoFisher Scientific, Massachusetts, USA), in order to stain acting filaments. Phalloidin was added to the samples after the secondary antibody, for 1–2 h at RT. Finally, cells were stained with Hoechst 33,258 (Life Technologies- Invitrogen, Carlsbad, CA,USA) (1:5000 in DPBS) for 5 min, washed again and then fixed with Mowiol (Sigma Aldrich, St. Louis, MO, USA) for epifluorescence and confocal microscopy.

### Microscopy and imaging

**Fig. 1:**
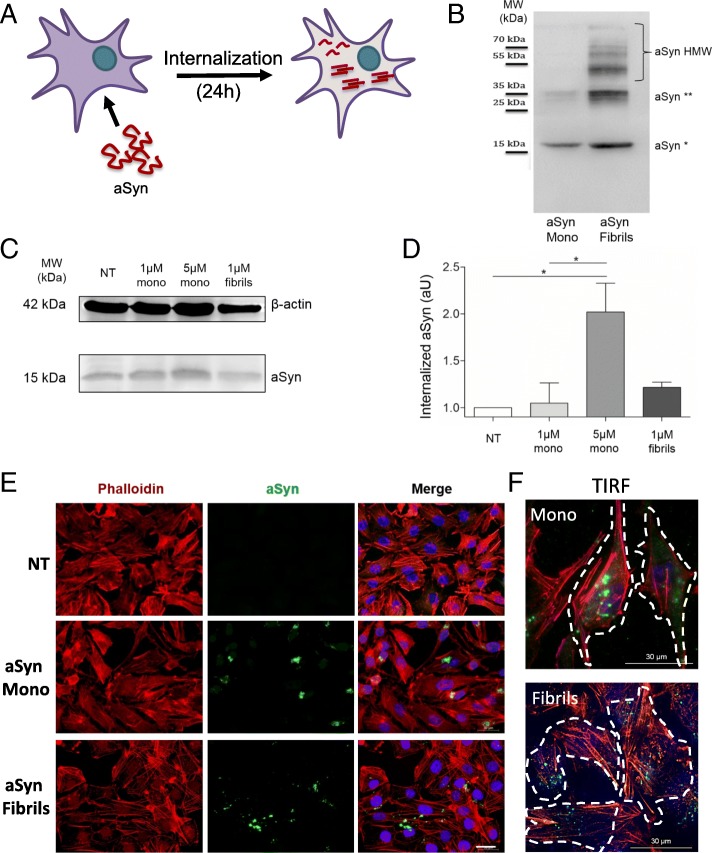
Recombinant aSyn monomers and fibrils are internalized by H4 cells. **a** Recombinant aSyn monomers (aSyn Mono) or fibrils (aSyn Fibrils) were added to the cell culture medium and incubated for 24 h. **b** SDS-PAGE and immunoblot analysis of the recombinant monomeric or fibrillar species of aSyn used in the experiments. The monomers show also the presence of a small fraction of dimers, as displayed by the faint band at 35 kDa. In the fibril preparation, one can observe the presence of higher molecular weight (HMW) species that are stable even on an SDS-PAGE. **c** Western blot (WB) of H4 cells after treatment with aSyn, confirming the internalization of aSyn monomers or fibrils as seen by the increase in the levels of aSyn in cells that were treated with monomers or fibrils (actin is used as a loading control). **d** Quantification of the immunoblots. Statistical test was performed using one-way ANOVA followed by Tukey’s post-hoc tests, *p < 0.01. **e** Epifluorescence microscopy of cells treated as indicated. Scale bar: 30 μm. **f** TIRF microscopy of cells treated with aSyn monomers or fibrils (green) and stained with Phalloidin (red) confirm intracellular localization of aSyn. Scale bar: 30 μm

Images in Fig. 1 and in Fig. 5 were acquired using a Leica Inverted Microscope DMI 6000 B (Leica, Wetzlar, Germany), using a 40× objective (HCX Pl Fluotar) or a 63× objective (HCX Pl Fluotar).

Total internal reflection (TIRF) microscopy was conducted on a Zeiss Axio Observer.Z1 microscope equipped with 405, 488, 561 and 639 nm lasers and an Evolve 512 EMCCD camera (Photometrics) and Zen Blue software, using 63× EC Plan-NEOFLUAR and 100× α Plan-APOCHROMAT objectives.

For the screening of Rab proteins, images were taken using an Olympus IX81-ZDC microscope system, with a 40× objective (FN 26,5). Sixty four images were randomly taken using the Olympus Scan^R Image Analysis Software in three independent experiments. Analysis of the aggregation patterns were performed qualitatively, by the experimenters, and the percentage of cells with inclusions was determined.

All the other images were acquired using a Airyscan Confocal Zeiss LSM800 with 40× or 63× oil immersion objectives.

For the colocalization analysis in Fig. 3, the Pearson’s R value was calculated via the use of Coloc2 plugin from ImageJ.

For the mean fluorescence intensity values in Figs. 8 was used Fiji (ImageJ) Software and GraphPad Prism for the statistical analyses and graph generation.

### NMR experiments

All NMR spectra were recorded on Bruker 600 MHz Avance III spectrometer, equipped with a cryogenically cooled triple resonance ^1^H(^13^C/^15^N) TCI probe. Experiments were recorded at 15 °C using protein samples dissolved in Buffer B supplemented with 10% D_2_O. For the ^1^H-^15^N HSQC experiments we used 16 scans, 1024 complex points (sweep-width of 16 ppm in the ^1^H dimension) and 256 complex points (sweep-width of 26 ppm in the ^15^N dimension). Sequence-specific assignments for the backbone of aSyn WT and aSyn A11P/V70P were transferred from previously published studies [39, 42]. Only unambiguously assigned, well resolved peaks were included in the analysis. The I/I_0_ ratios obtained for aSyn WT and aSyn A11P/V70P, in absence and presence of SUVs were plotted as a function of the protein sequence to obtain the intensity perturbation profiles [33]. Mean weighted chemical shifts displacements (MWΔCS) for ^1^H-^15^N were calculated as [(Δδ^1^H)^2^ + (Δδ^15^N/10)^2^]^1/2^.

Acquisition and processing of NMR spectra were performed using TOPSPIN 3.2 (Bruker Biospin). 2D spectra analyses were performed with CCPN.

### SUV preparation

SUVs were prepared from a molar ratio of 1:1 of Coagulation Reagent I containing DOPE:DOPS:DOPC (5:3:2 *w*/w) and DOPC (both Avanti Polar Lipids Inc., USA) dissolved in chloroform yielding a final molar ratio of DOPE:DOPS:DOPC (5:3:12 w/w). The lipid solution formed a thin film under evaporation of the solvent with nitrogen gas and was further dried by lyophilization under vacuum. The dried phospholipids were dissolved in MES buffer (20 mM MES, 100 mM NaCl, pH 6.5) and underwent several cycles of freeze-thawing and water bath sonication until the solution became clear. The size distribution was also checked by DLS. For the NMR experiments a SUV stock solution of 85 mM (6.6% *w*/*v*) in respect to the monomers was used.

### Software and statistical analyses

Colocalization in ICC samples was measured by using ImageJ software and Pierson’s Coefficient was calculated and detailed colocalization analysis were performed with the use of Coloc2 Plugin from Fiji (ImageJ software). Figures were composed with CorelDRAW X8 (Corel Corporation, Ottawa, Canada) or with Microsoft Power Point (Microsoft Corporation).

Statistical analysis was performed using Microsoft Excel (Microsoft Corporation) and GraphPad PRISM 5 (GraphPad Software, San Diego, CA, USA). Images were processed with ImageJ V1.41, NIH, USA and/or CorelDRAW X8 (Corel Corporation, Ottawa, Canada).

Statistical tests performed were Student’s-two-tailed t-test, one-way-Analysis of Variance (ANOVA) and repeated-measures ANOVA for grouped analysis, followed by Tukey’s post-hoc tests for multiple comparison.

Data were expressed as mean ± SEM and a 0.5% general significance level was defined, with significance levels as follows: *: *p* < 0.05; **: *p* < 0.01; ***: *p* < 0.001.

## Results

### aSyn is internalized and forms intracellular inclusions

In order to investigate the molecular determinants of aSyn internalization, we compared the behaviour of two distinct forms of recombinant aSyn (monomers or fibrillar aggregates, hereafter named fibrils) (Fig. 1a). aSyn monomers and fibrils were generated as described in Methods, and the species were characterized by Transmission Electron Microscopy (TEM) [62] and by SDS-PAGE (Fig. 1b). The preparation of aSyn monomers showed also the presence of a smaller amount of dimers, as illustrated by the band at ~ 35 kDa. Due to contrasting studies reporting the ability of monomeric aSyn to passively enter cells, we tested two different concentrations of this form (1 μM and 5 μM), and 1 μM of fibrils of aSyn (calculated based on the initial concentration of monomeric aSyn). The internalization of aSyn was analysed by immunoblotting and by immunocytochemistry (ICC) after 24 h of incubation with cells. Immunoblot analysis revealed that both aSyn monomers and fibrils were internalized, given the increase in the levels of aSyn in treated cells (Fig. 1c and d). Furthermore, microscopy analysis (confocal and TIRF microscopy) demonstrated that, in cells exposed to monomeric aSyn, the protein accumulated in distinct perinuclear puncta, whereas in cells exposed to fibrils aSyn accumulated in larger cytosolic inclusions (Fig. 1e-f).

### aSyn interacts with the plasma membrane and accumulates as high molecular weight species

**Fig. 2:**
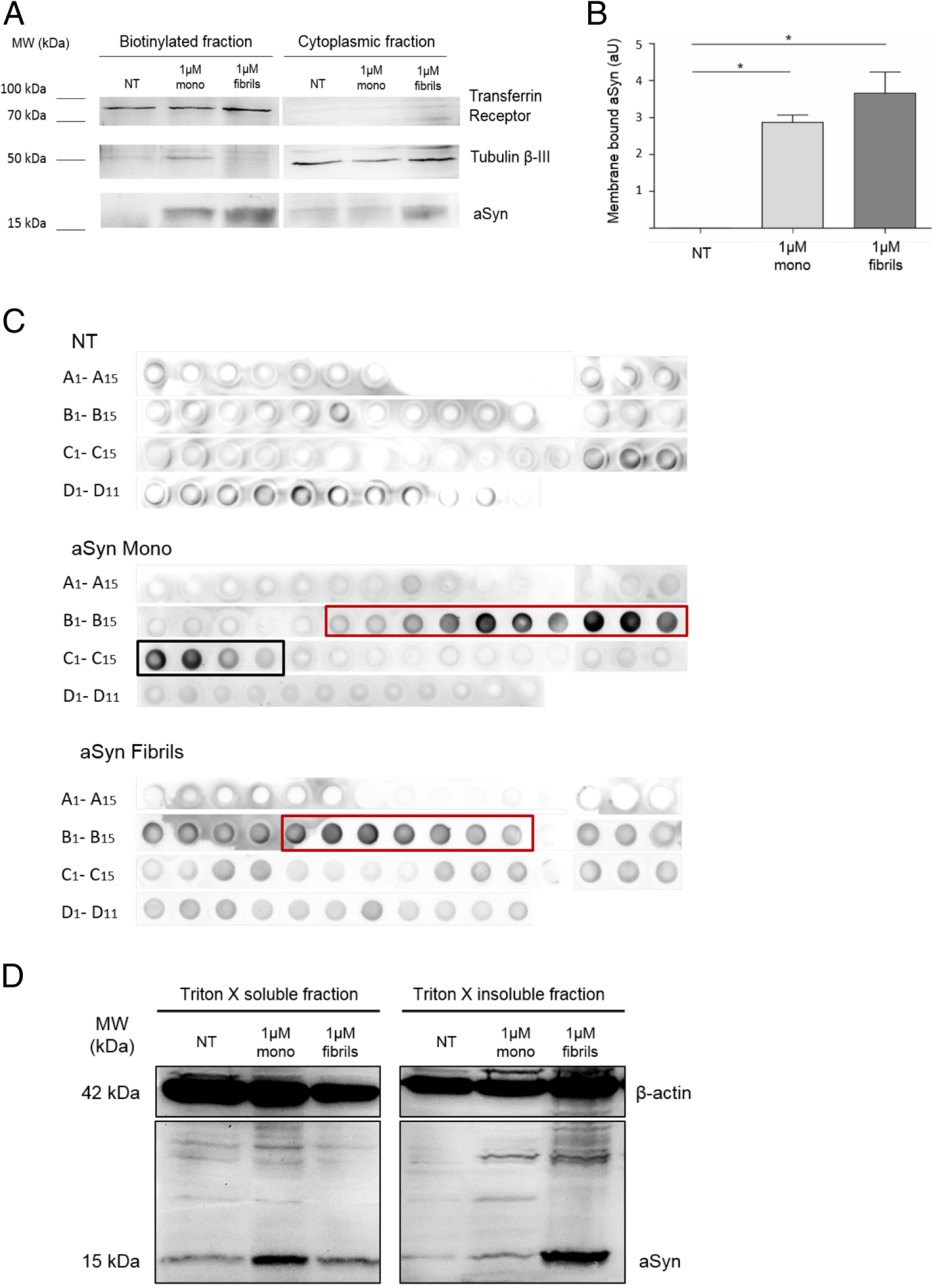
aSyn associates with membranes and forms high molecular weight species during the internalization process in H4 cells. **a** Immunoblot of the biotinylation assay of cells treated with aSyn monomers or fibrils (tubulin is used as a loading control). **b** Quantification of the levels of aSyn present in the biotinylated fraction (membrane-associated aSyn). Statistical test was performed using one-way ANOVA followed by Tukey’s post-hoc tests, *p < 0.01 **c** Dot blot of the size exclusion chromatography fractions of lysates of untreated cells, cells treated with 1 μM of aSyn monomers, and cells treated with 1 μM of aSyn fibrils. The black box highlights monomeric aSyn, while the red boxes highlight the presence of high molecular weight species of aSyn. **d** Triton X-100 fractionation, with the soluble (left panel) and the insoluble fractions (right panel), treated as described

Based on previously reported aSyn binding to membranes, we hypothesized that the internalization of aSyn might involve an interaction with the plasma membrane. In order to test this, we performed a cell surface biotinylation assay in cells treated with aSyn. aSyn was found in the biotinylated fraction treated with either monomers or fibrils, indicating that extracellular aSyn interacted with the plasma membrane (Fig. 2a and b), and then accumulated within the cell in punctae (Fig. 1e-f).

To further investigate the biochemical nature of the intracellular aSyn, we performed size exclusion chromatography (SEC) of cell lysates, and then dot blot analysis of the various fractions collected. Recombinant aSyn monomers were assessed by SEC in parallel, in order to establish the elution profile of this form of aSyn (Additional file 1: Figure S1A and S1B).

As expected, dot blot analysis of non-treated cells (NT) showed low signal, given the lower levels of aSyn (Fig. 2c). In cells treated with aSyn monomers, we detected the protein in fractions C1 to C4 (black box, Fig. 2c) as reported in the chromatogram (Additional file 1: Figure S1B). We also detected aSyn in the final B fractions (from B6 to B15, red box), indicating the presence of higher molecular weight aSyn species as well (Fig. 2c). Such species were also found in cells treated with aSyn fibrils (fractions B5 to B11, red box), but not monomeric species of aSyn (Fig. 2c), suggesting that the species accumulating in the cells were biochemically distinct depending on the species of aSyn added to the cells.

To further confirm the biochemical differences observed, we performed differential fractionation of the cell lysates using Triton X-100. Immunoblot analysis showed higher levels of Triton X-100-soluble aSyn in cells treated with monomers, and higher levels of Triton X-100-insoluble aSyn in cells treated with fibrils, consistent with the results of the SEC analysis (Fig. 2d, left side). Interestingly, we also detected the formation of high molecular weight aSyn species in cells treated with monomeric aSyn, suggesting that, upon internalization, aSyn monomers start to aggregate (Fig. 2d, right side).

Taken together, these results suggest that both monomeric and fibrillar aSyn enter cells and accumulate in aggregated, high molecular weight species.

### aSyn partially colocalizes with Rab5A and Rab7

Next, we performed a microscopy-based screen of mammalian Rab proteins in order to identify the interplay between aSyn and the trafficking pathways. The experiment consisted of treating cells overexpressing each individual mammalian Rab protein (fused to EGFP: Rab-GFP) with aSyn monomers or fibrils in order to assess (i) the effect of aSyn on the subcellular distribution of the Rab proteins, and (ii) the effect of each Rab protein on the subcellular distribution of aSyn. From the screen, we selected a set of Rab proteins whose localization was altered, or that colocalized with aSyn (Additional file 2: Table S1). Of these, we selected Rab4A, Rab5A and Rab7 since they resulted in the strongest phenotype. Interestingly, the Rab proteins identified in the screen are compatible with a hypothesis that aSyn is internalized via an active endocytic mechanism that then sorts the protein into vesicular compartments, such as endosomes and lysosomes [57]. Therefore, we next focused our study on the these Rab proteins.

**Fig. 3:**
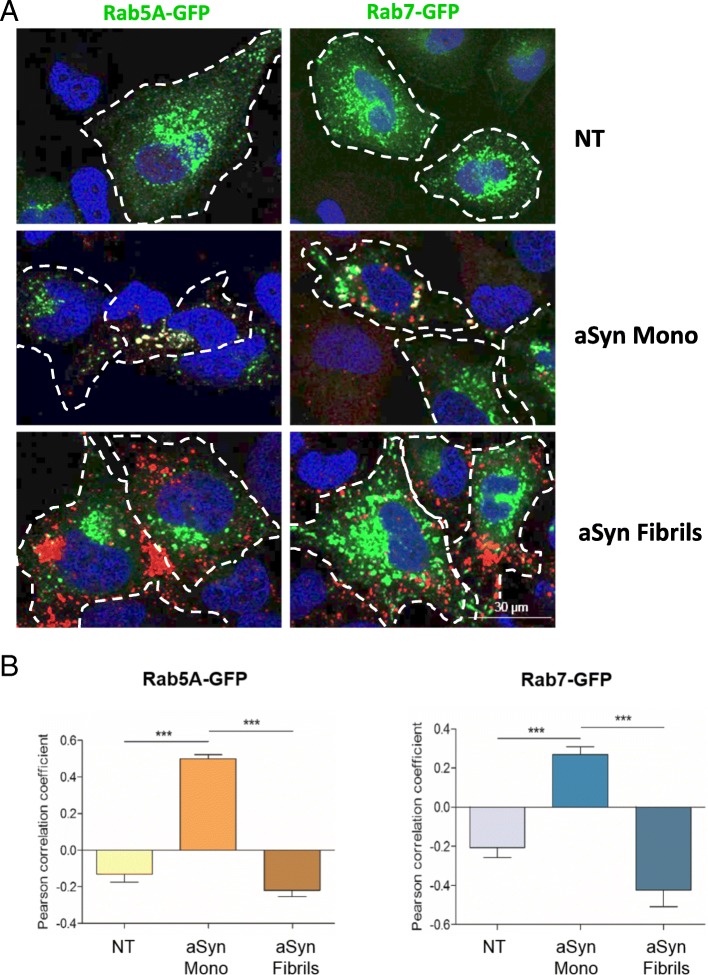
aSyn partially colocalizes with Rab5A-GFP and Rab7-GFP in H4 cells. **a** ICC of cells transfected with Rab5A-GFP (right side of the panel) or with Rab7-GFP (left side) and then treated with 1 μM of aSyn monomers or fibrils. **b** Pearson correlation coefficient reveals colocalization of aSyn and Rab5A, and of aSyn and Rab7 in cells treated with aSyn monomers, but not with fibrils. Scale bar: 30 μm

First, we assessed the degree of colocalization of aSyn and Rab5A-GFP, or Rab7-GFP, in cells treated with aSyn monomers or fibrils (Fig. 3a). The colocalization was quantified using the Coloc2 plugin of ImageJ Software (Fig. 3b). In cells treated with aSyn monomers, we observed a strong colocalization between aSyn (in red) and Rab5A-GFP vesicles (in green) (Fig. 3a, left column, central panel), as well as a partial, although weaker, colocalization with Rab7-GFP (Fig. 3a, right column, central panel). Interestingly, the colocalization was not observed when cells were treated with aSyn fibrils. This supports the idea that the internalization and sorting of aSyn monomers and fibrils is different, as one might expect given their distinct biochemical properties.

### aSyn form inclusions in Rab4A-positive compartments

**Fig. 4:**
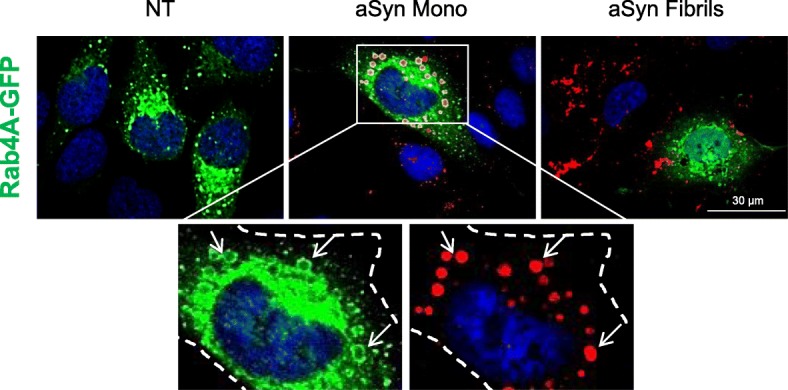
aSyn accumulates in Rab 4A-positive vesicles. ICC on H4 cells transfected with Rab4A-GFP and treated with 1 μM of aSyn monomers or fibrils. Inset: zoom and separated channels of Rab4A-GFP aSyn. Arrows point to the large inclusions aSyn (in red, panel on the right) matching with the GFP-positive Rab4A vesicles (in green, panel on the left). Scale bar: 30 μm

Next, we examined the interplay between Rab4A and aSyn. We found no effect on the distribution of Rab4A-GFP in cells treated with aSyn fibrils. Likewise, we also found no colocalization between Rab4A-GFP and aSyn in those cells (Fig. 4, right panel). In contrast, when Rab4A-GFP-expressing cells were treated with aSyn monomers, we observed a prominent increase in the size of endosomes, as well as a massive internalization of aSyn that accumulated in compartments surrounded by large, abnormal rings of Rab4A (Fig. 4, central panel on the top and lower panels). This change in the size of early endosomes suggested that exposure to aSyn monomers altered the normal biology of Rab4A and, therefore, the endosome-related trafficking processes.

### Membrane binding properties are essential for the internalization of aSyn

**Fig. 5:**
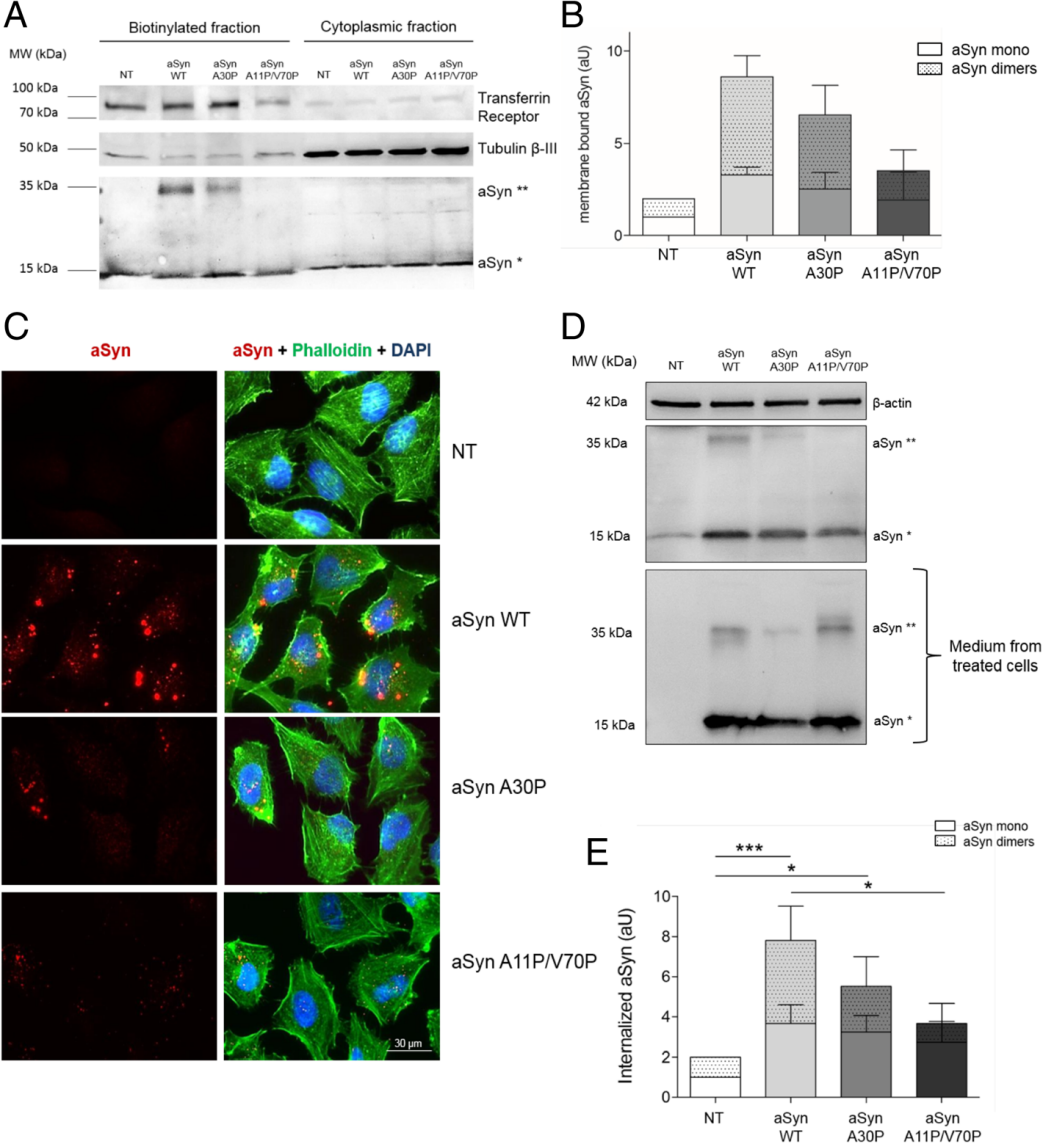
Membrane binding is essential for the aSyn internalization and inclusion formation. **a** and **b** Immunoblot and quantification of the comparison of WT aSyn and two mutants (A30P and A11P/V70P) with different membrane-binding properties using a biotinylation assay. Dotted bars refer to the band corresponding to aSyn dimers (aSyn**), and clear bars refer to aSyn monomers (aSyn*). **c** ICC and **d** immunoblotting of non-treated (NT) cells, or cells treated with WT, A30P, or A11P/V70P aSyn for 24 h. Scale bar: 30 μm. **e** Quantification of the immunoblot in panel D. Dotted bars refer to the band corresponding to aSyn dimers (aSyn**), and clear bars refer to aSyn monomers (aSyn*). Statistical tests were performed using one-way ANOVA with repeated-measures for grouped analysis, followed by Tukey’s post-hoc tests. Data were expressed as mean ± SEM and a 0.5% general significance level was defined, with significance levels as follows: *: *p* < 0.05; **: p < 0.01; ***: *p* < 0.001. Statistical significance is indicated with the symbol “#” for the monomers, “+” for the dimers, and “*” for the combination between monomers and dimers

Based on the stronger effects of aSyn monomers, we decided to focus on the effects of monomeric aSyn. To investigate whether intrinsic aSyn properties affected the internalization of the protein, we took advantage of different aSyn mutants that have different membrane binding abilities. Specifically, we used WT aSyn, the aSyn A30P familial mutant, known to display weaker binding to membranes [4, 12, 29, 30, 61], and the artificial mutant (A11P/V70P) designed to severely impair membrane binding [8, 10]. First, we performed membrane biotinylation assays with the different mutants, and detected a clear trend in the amount of protein present in the biotinylated fractions that reflected the different membrane binding properties of the aSyn mutants (aSyn A30P and aSyn A11P/V70P) (Fig. 5a and b). In particular, we detected a consistent trend in the levels of aSyn dimeric species in the biotinylated fraction, suggesting that membrane binding is important for dimerization and aggregation of aSyn.

Next, we tested the ability of the two aSyn mutants to enter cells and accumulate in intracellular inclusions, via immunocytochemistry (ICC) approach. We observed a significant reduction in the accumulation of aSyn in inclusions in cells treated with A30P or A11P/V70P aSyn mutants when compared with cells treated with WT aSyn (Fig. 5c), consistent with a difference in the internalization of the mutants. We further quantified the internalization of the different variants of aSyn using immunoblot analysis. We confirmed a strong difference in the internalization of the various mutants (Fig. 5d and e), although the amount of aSyn present in the medium was identical (Fig. 5d).

Taken together, these results suggest that membrane binding is essential for the internalization and, therefore, for the formation of intracellular aSyn inclusions.

### aSyn A11P/V70P is unable to bind membranes

To test the contribution of different aSyn regions towards aggregation and membrane binding, several artificial mutants, such as the aSyn A11P/V70P have been designed [9, 11]. First, we validated the effect of the A11P/V70P mutation on the biding of aSyn to small unilamellar vesicles (SUVs) (5:3:12 mixture of DOPE:DOPS:DOPC) using NMR spectroscopy (Additional file 3: Figure S2). While WT aSyn interacted with DOPE:DOPS:DOPC SUVS, as previously described [22, 34] (Additional file 4: Figure S3A, panel on the left), the aSyn A11P/V70P mutant displayed a drastic reduction (more than 80%) of the signal broadening starting from residue 11 (where the first Pro mutation is located) until residue 140 (Additional file 4: Figure S3A, panel on the right), confirming the impairment in membrane binding.

Next, we tested the effect of aSyn A11P/V70P in the cellular model, in comparison to WT aSyn, and also in the presence or absence of Rab4A-GFP, as we had observed increased internalization of aSyn in H4 cells. Using immunoblot analysis and ICC, we found that the internalization A11P/V70P aSyn was negligible when compared to that of WT aSyn (Additional file 4: Figure S3B-D). Importantly, we did not detect internalization when we increased the concentration of A11P/V70P aSyn or when we overexpressed Rab4A-GFP, conditions which significantly increased the levels of intracellular WT aSyn (Additional file 4: Figure S3B-D).

### The endocytic pathway is involved in the internalization of aSyn

**Fig. 6:**
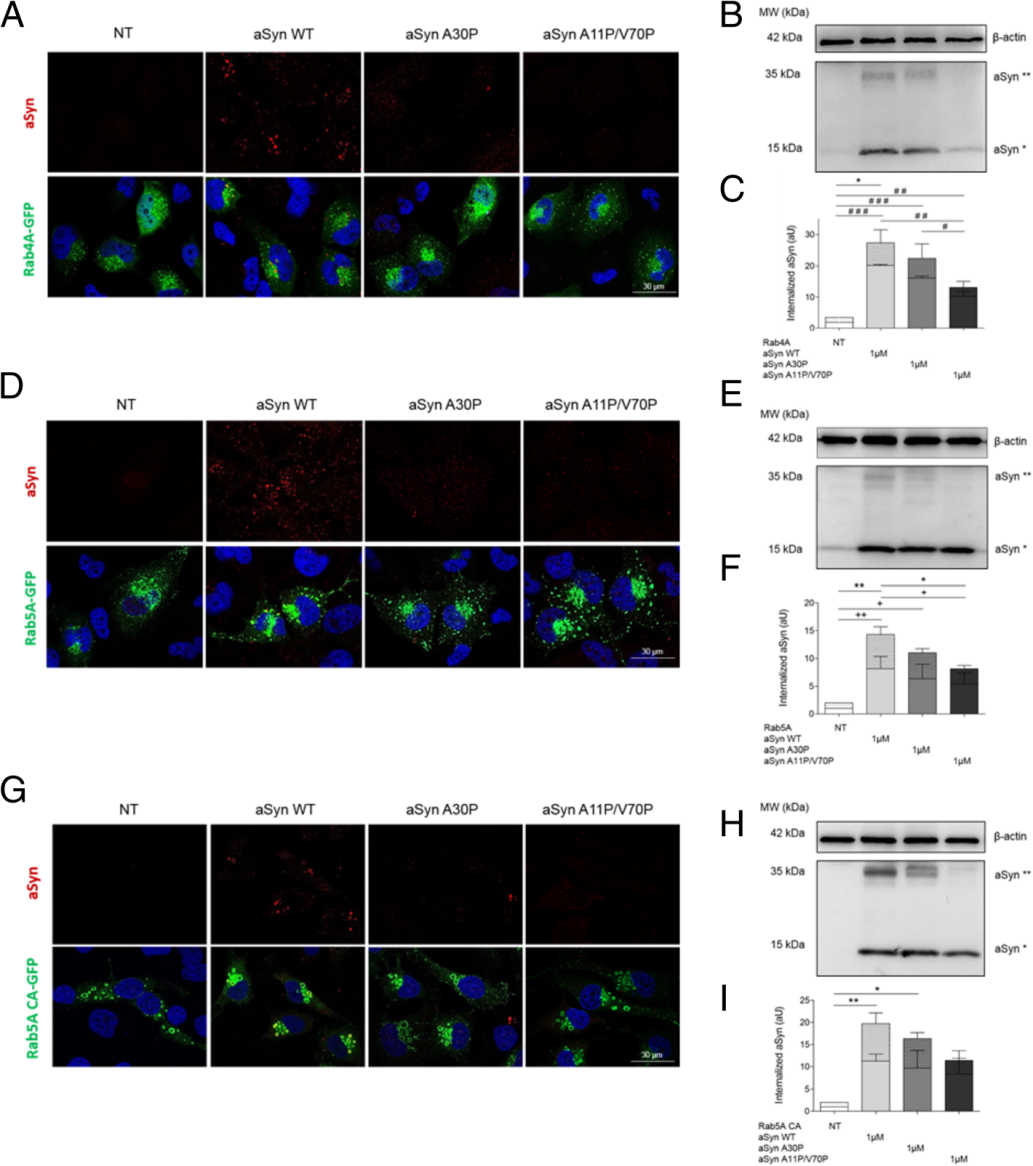
The A30P and A11P/V70P aSyn mutants are less internalized than WT aSyn. **a** ICC and **b** Immunoblotting of cells transfected with Rab4A–GFP and treated as in experiments shown in Fig. 5. **d** and **e** ICC and Immunoblotting of cells transfected with Rab5A-GFP and treated as above. **g** and **h** ICC and Immunoblotting of cells transfected with Rab5ACA-GFP (constitutively active) and treated as above. **c**, **f** and **i** Quantifications of the immunoblots in panels **b**, **e** and **h**. Dotted bars refer to the band corresponding to aSyn dimers (aSyn**), and clear bars refer to aSyn monomers (aSyn*). Statistical tests were performed using one-way ANOVA with repeated-measures for grouped analysis, followed by Tukey’s post-hoc tests. Data are expressed as mean ± SEM and a 0.5% general significance level was defined, with significance levels as follows: *: *p* < 0.05; **: *p* < 0.01; ***: *p* < 0.001. Statistical significance is indicated with the symbol “#” for the monomers, “+” for the dimers, and “*” for the combination between monomers and dimers. Scale bar: 30 μm

In order to investigate whether mutants with different membrane binding properties altered the internalization and intracellular fate of internalized aSyn, we used cells overexpressing Rab4A-GFP, Rab5A-GFP, or a constitutively active (CA) mutant of Rab5A-GFP. As described above, WT aSyn was readily internalized and accumulated in Rab4A-GFP-positive vesicles. In contrast, the internalization of the artificial A11P/V70P aSyn mutant was strongly impaired. Curiously, the PD-associated mutant A30P displayed an intermediate phenotype (Fig. 6a-c).

Interestingly, the levels of internalized aSyn (monomers and dimers) were higher in cells expressing these Rab proteins than in naïve cells (shown above in Fig. 5d-e), suggesting that increased levels of Rab4A altered the dynamics of internalization and dimerization of aSyn. The same trend was observed in in cells overexpressing Rab5A-GFP indicating, once again, that stimulation of the early steps of endosome formation increased the internalization of aSyn, as long as the membrane binding properties of the protein are preserved (Fig. 6d-f). In cells overexpressing Rab5A, we also observed an increase in the levels of aSyn dimeric species.

Finally, to confirm the functional involvement of Rab5A on the internalization of aSyn, we used a mutant in which the GTPase activity is deregulated, resulting in permanent activation - constitutively active mutant - Rab5ACA-GFP (Fig. 6g-i). In cells expressing this mutant Rab5A, we found overall higher levels of aSyn internalization, further confirming the role of the endocytic pathway in the internalization of aSyn.

### Rab7 sorts aSyn for degradation and reduces its intracellular accumulation

**Fig. 7:**
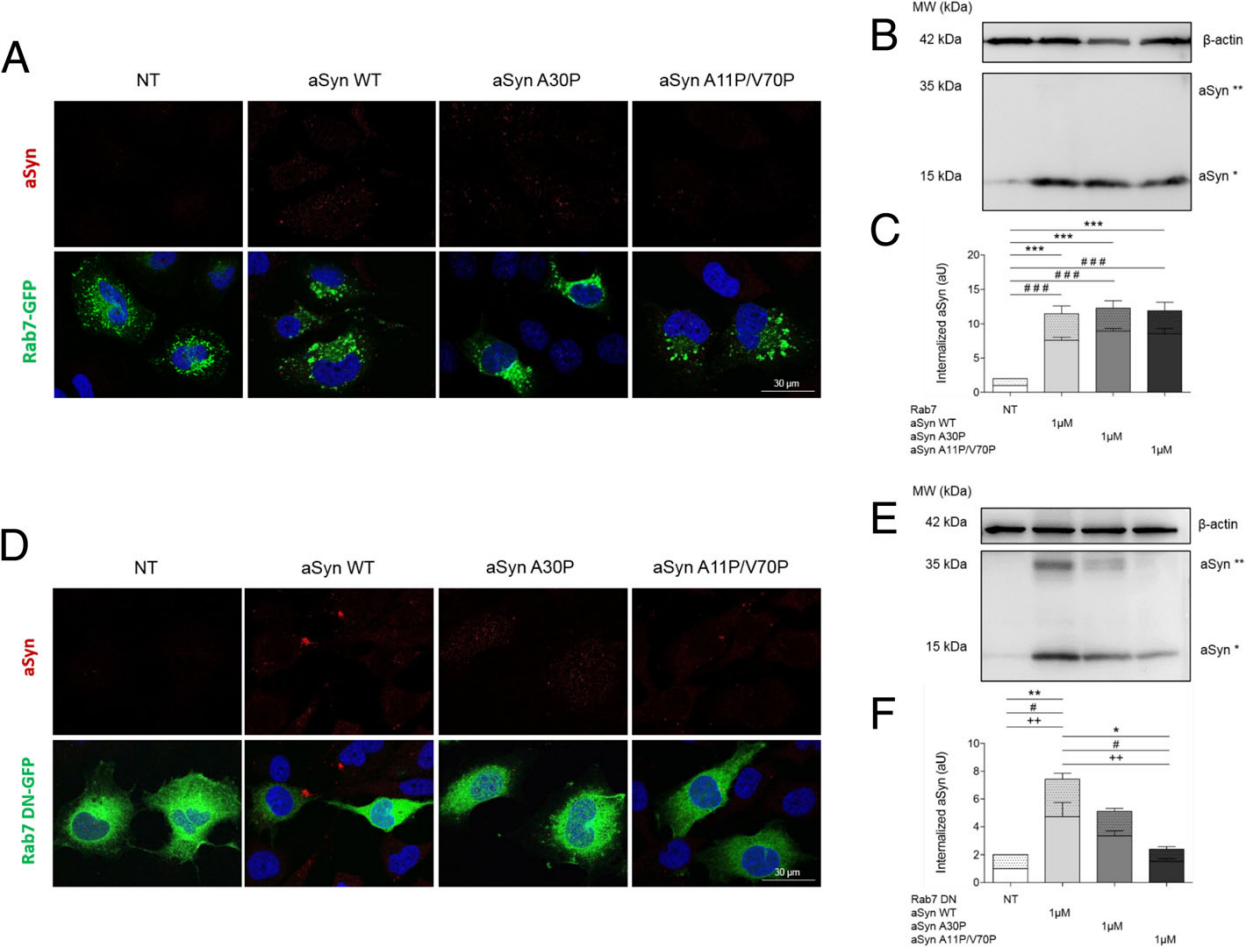
Rab7 reduces the formation of dimers in cells treated with WT aSyn monomers. **a** ICC and **b** Immunoblotting of H4 cells transfected with Rab7-GFP and treated as described above. **d** and **e** The overexpression of the Rab7 dominant negative (DN) does not affect the degradation of the internalized aSyn. **c** and **f** Quantifications of the immunoblots in panels B and E. Dotted bars refer to the band corresponding to aSyn dimers (aSyn**), and clear bars refer to aSyn monomers (aSyn*). Statistical tests were performed using one-way ANOVA with repeated-measures for grouped analysis, followed by Tukey’s post-hoc tests. Data were expressed as mean ± SEM and a 0.5% general significance level was defined, with significance levels as follows: *: *p* < 0.05; **: *p* < 0.01; ***: *p* < 0.001. The significance is shown with the symbol “#” for the monomers, with the symbol “+” for the dimers and with the symbol “*” for the sum between monomers and dimers. Scale bar: 30 μm

Next, we investigated the intracellular fate of internalized aSyn along the endocytic pathway by using Rab7-GFP as a marker. Cells expressing Rab7-GFP were treated with WT, A30P, or A11P/V70P aSyn mutants, and analysed by ICC and immunoblotting, as described above. Surprisingly, we found that the internalization of aSyn, and the formation of dimers, was significantly reduced in cells overexpressing Rab7, and that there were no differences in internalization between WT aSyn or the two mutants. (Fig. 7a-c).

We hypothesized that this effect could be due to the sorting of aSyn for degradation in the lysosome, due to increased stimulation of the lysosomal pathway by overexpression of Rab7. To test this, we repeated the experiment in cells expressing a dominant negative (DN) mutant of Rab7 (Rab7DN-GFP) that impairs its activity. Interestingly, we found that the internalization and dimerization of aSyn was restored to the initial levels, suggesting that the Rab7DN mutant blocked the sorting of aSyn to the lysosome (Fig. 7d-f).

### The internalization of aSyn is mediated by dynamin

Next, we investigated the mechanism involved in the internalization of aSyn by using two well established chemical blockers of endocytosis: PitStop2 (PitStop) and Dyngo 4A (Dyngo).

Pitstop is a selective inhibitor of clathrin-mediated endocytosis (CME) [50, 53], while Dyngo blocks all dynamin-dependent endocytic mechanisms [40].

Naïve cells or cells overexpressing Rab4A-GFP were treated with each of the two compounds for 30 min prior to the treatment with aSyn monomers, and were then incubated together with aSyn for 24 h and processed for ICC or immunoblotting, as described above.

In naive cells, Dyngo efficiently blocked the internalization of aSyn (Additional file 5: Figure S4A). Yet, the opposite effect was observed using PitStop, which increased the accumulation of intracellular aSyn (Additional file 5: Figure S4B-D).

**Fig. 8:**
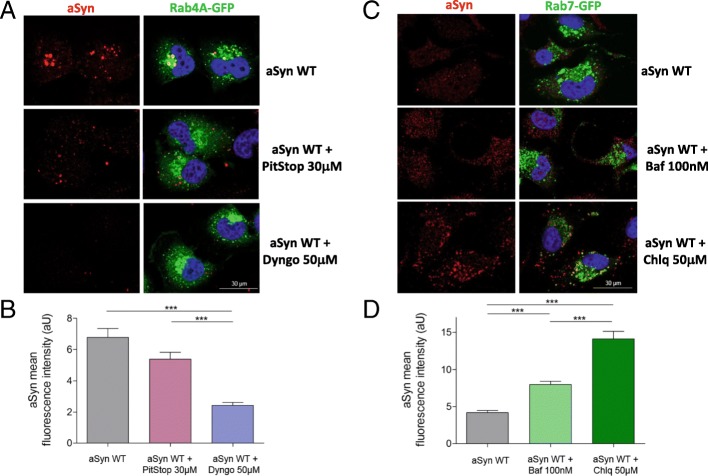
Blocking endocytosis and autophagy modulates the internalization and degradation of aSyn. **a** ICC of Rab4A-GFP expressed in H4 cells treated with 1 μM aSyn monomers and with vehicle, PitStop 30 μM or with Dyngo 50 μM. Both PitStop and Dyngo are inhibitors of the endocytic processes. **b** Quantification of the aSyn mean fluorescence intensity in the three conditions. **c** ICC of Rab7-GFP-expressing cells treated with 1 μM aSyn monomers and with vehicle, Bafilomycin 100 nM (Baf 100 nM), or with Chloroquine 50 μM (Chlq 50 μM). Bafilomycin and Chloroquine are inhibitors of lysosomal acidification and, therefore, autophagy-lysosome pathway (ALP). (D) Quantification of the aSyn mean fluorescence intensity in the three conditions. Scale bar: 30 μm

Similarly, in cells overexpressing Rab 4A, Dyngo prevented the internalization and accumulation of aSyn in Rab4A-surrounded vesicles. In contrast, PitStop failed to produce a significant effect (Fig. 8a-b).

### Internalized aSyn is degraded by lysosomes

To investigate the fate of internalized aSyn, we tested whether blocking lysosomal and autophagic function would affect the levels of internalized aSyn. We treated cells expressing Rab7-GFP with bafilomycin A1 or chloroquine for 30 min, and then added monomeric aSyn.

Cells were then incubated for 24 h, and then processed for ICC analysis. We confirmed that blocking lysosomal acidification and consequently autophagy inhibited the degradation of internalized aSyn and led to its accumulation, possibly in late endosomes and lysosomes (Fig. 8c-d). Identical results were obtained in naïve cells (Additional file 5: Figure S4, A-D). Interestingly, treatment with chloroquine resulted in stronger accumulation of aSyn than that observed with Bafilomycin A1, consistent with chloroquine being more potent inhibitor then bafilomycin A1.

Taken together, our results suggest that aSyn is internalized via dynamin-mediated endocytosis and trafficked via the endocytic pathway and then sorted and targeted for degradation via the autophagy-lysosomal system.

## Discussion

A key premise for the prion-like spreading hypothesis of aSyn pathology in PD and other synucleinopathies is that aSyn assemblies are taken up by a recipient cell, are sorted, and then seed the aggregation of endogenous aSyn, thereby propagating pathology [6, 7]. Nevertheless, the precise molecular mechanisms involved are still elusive. Most studies have focused on the understanding of the mechanisms through which aSyn can be released from cells. However, present understanding of the uptake and fate of internalized aSyn are even more limited. Here, we focused on the initial steps of this complex process, and investigated how membrane binding properties might affect the internalization and processing of aSyn in the cell. Using a simple cell-based paradigm, we established that both monomeric and fibrillar aSyn proteins can interact with the plasma membrane and be internalized, accumulating in high molecular weight species.

We also investigated the role of aSyn membrane binding on internalization by using mutants that are known to affect the membrane binding properties of aSyn. The PD-associated mutant A30P displays reduced membrane interactions and reduced internalization, when compared to WT aSyn. Structurally, the substitution of the alanine by the proline disrupts the α-helical domains formed in the N-terminal and central regions of aSyn, thereby affecting the ability of the protein to interact and bind to membranes. Consistently, we found that the artificial mutant A11P/V70P, designed to disrupt membrane binding [9, 11], indeed disrupts the N-terminal region and compromises membrane binding, thereby impairing internalization of aSyn. Interestingly, it has been widely suggested that the aSyn A30P mutant may cause PD by affecting at least partially different cellular pathways that those affected by WT aSyn. Our study is consistent with this idea, and forms now the basis for in vivo investigations of the prion-like spreading ability of this mutant, which has not been documented, since the studies performed thus far focused on WT aSyn [57–60].

Importantly, our findings suggest that one cannot dismiss that, even monomeric aSyn might be sufficient to initiate the process of aggregation and therefore, the spreading of aSyn pathology. Only two studies have reported the internalization of aSyn monomers and the involvement of the endocytic pathway, although the purpose and the readouts used in those studies were different [1, 58]. Interestingly, a comparable study reported consistent results regarding tau, suggesting that extracellular monomeric tau is sufficient to initiate the spreading of tau pathology [41]. However, one caveat of our study is the concentration of aSyn used, which is higher than that used in several other studies [30, 61]. Nevertheless, our study establishes proof of concept that, perhaps a local and even just temporary increase in the concentration of aSyn, caused for example by dying cells in the brain, might lead to the release of monomeric aSyn that could be internalized by neighbouring cells, thereby initiating the spreading of pathology.

Using selective inhibitors of the endocytic pathways, such as Dyngo and PitStop, we demonstrate that aSyn is internalized in a dynamin-dependent process, but not through CME. It is possible that when clathrin-mediated mechanisms are blocked (PitStop-related effects), the clathrin-independent processes (blocked by Dyngo) which are presumably involved in the internalization of aSyn are exacerbated. In this way, a larger amount of aSyn can be internalized in naïve cells (Additional file 5: Figure S4). Additional studies are required to further dissect the molecular mechanisms involved in aSyn endocytosis.

In an unbiased screen of Rab proteins, we found that, once internalized, aSyn partially colocalizes with Rab5A and Rab7, suggesting that the endosomal pathway is involved in the sorting and processing of aSyn. Furthermore, we demonstrated that Rab4A modulates the internalization of aSyn, and surrounds the internalized protein. Both Rab4A and Rab5A are localized in the early endosomes, contributing to the recycling/transport of proteins to the plasma membrane, respectively. Rab7 is localized in the late endosomes, lysosomes and phagosomes, contributing to the fusion of late endosome with lysosome [27]. Recent studies suggest that, even when different Rabs localize in the same compartment, they can occupy distinct membrane microdomains with different functions, called Rab domains. These are dynamic structures but, interestingly, do not mix significantly over time [52]. One of the possible explanations for this segregation mechanism is that it could be, at least in part, mediated by effector proteins and their association with additional molecules in the organelle membranes [52]. Consistently, a recent study showed that Rab4A is essential for the recruitment of adaptor proteins to the tubular subdomain of the early endosomal membrane, orchestrating a signalling cascade that initiates the formation of a sorting platform from which multiple classes of vesicular carriers emerge [18]. Based on the perturbation of the morphology of Rab4A vesicles due to the interaction with aSyn, we hypothesize that this particular GTPase cascade nucleated by Rab4A is actively involved into the internalization of aSyn. Future studies should focus on the precise molecular link between Rab4A and aSyn, and with other potential effector proteins.

Our data are also consistent with the idea that aSyn aggregation may be favoured by the low pH - that is reported to vary between 5.0 and 6.0 - in endosomes [14], as well as by molecular crowding due to the presence of acidic endosomal proteins. It is also possible that aSyn aggregation occurs due to problems in the late endosome-lysosomal compartments, which we found to be involved in the degradation of aSyn. Thus, impaired autophagic degradation of aSyn could lead to the accumulation of aggregation-competent species [19].

**Fig. 9:**
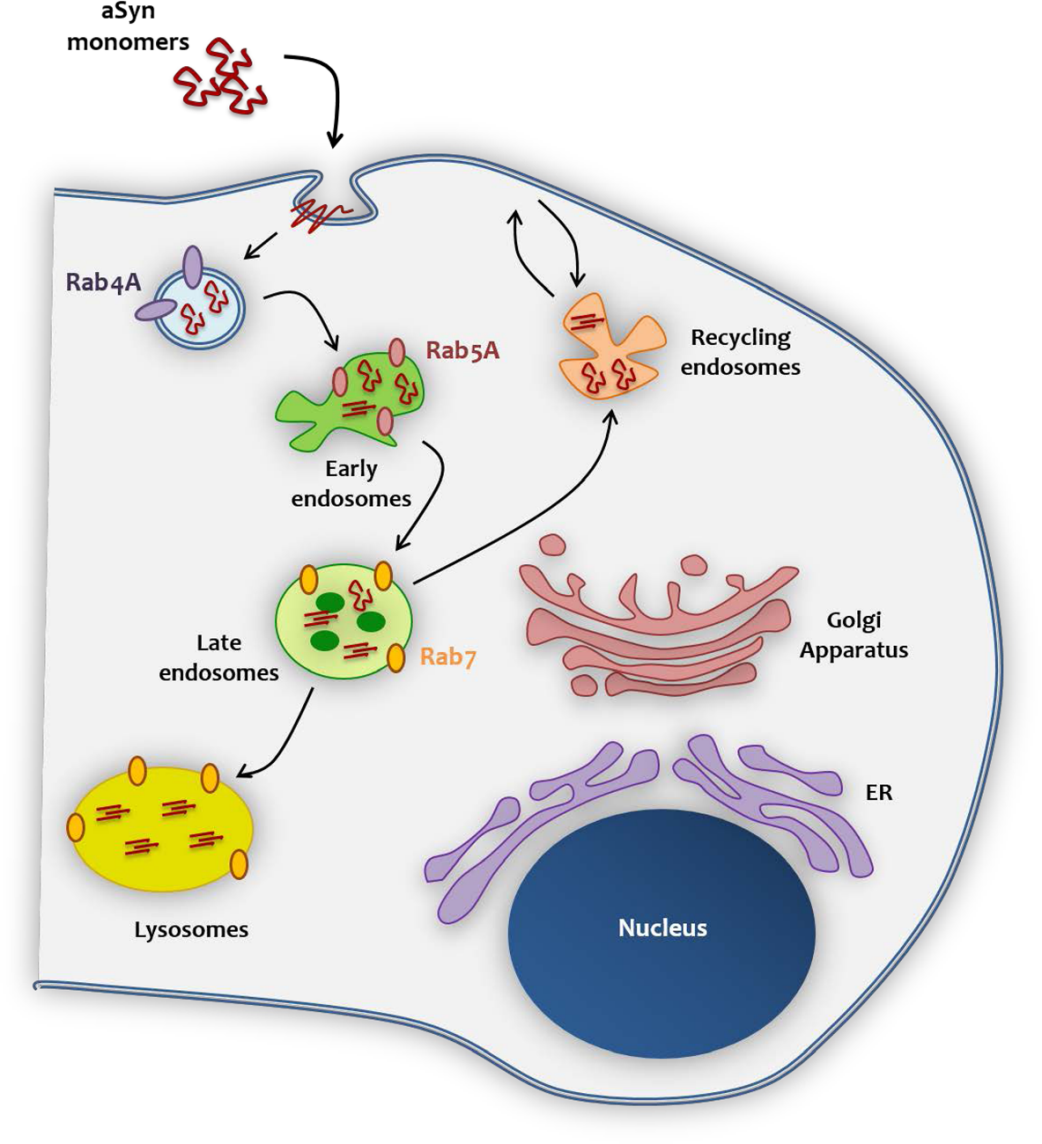
Model for the proposed mechanism of aSyn internalization and interaction with the membrane and trafficking machinery. After interacting with the plasma membrane, aSyn monomers are internalized through the endocytic pathway. Rab4A plays an important role in the sorting of aSyn and on the transport from/to the plasma membrane. aSyn is then found in the early endosome and, it is possible that during the progress from early to late endosome, due to acidification of the environment, aSyn monomers start to aggregate and form high molecular weight species. Afterwards, late endosomes fuse with lysosomes, where aSyn can be degraded. It is possible that, during any of those steps, aSyn is released to the cytoplasm and to the extracellular milieu, leading to its potential accumulation and spreading of pathology

Our data enable us to propose a model for the internalization of aSyn, based on the interaction with trafficking machinery components (Fig. 9). After interacting with the membrane, aSyn monomers are internalized through the endocytic pathway, where Rab4A plays an important role on the protein sorting and on the transport from/to the plasma membrane. aSyn is thereafter sorted to the early endosome (in colocalization with Rab5A). It is possible that during the progress from early to late endosome (where it colocalizes with Rab7), due to pH acidification, aSyn monomers start to oligomerize, and to assemble in high molecular weight species, that can then escape to the cytoplasm, leading to its accumulation and facilitating the spreading of aSyn pathology.

## Conclusions

In total, our study emphasizes the importance of membrane binding for the internalization of aSyn and highlights the fundamental role of Rab proteins in the internalization, sorting, and processing of aSyn, suggesting that targeting specific Rab proteins and/or specific intracellular trafficking components might prove to be valuable targets for modulating the spreading of aSyn pathology and, consequently, disease progression in PD and other synucleinopathies.

## Additional files

Additional file 1: **Figure S1.** Characterization of recombinant aSyn monomers. (A) Fractions collected upon protein separation on Superose 6 10/300 size exclusion column. (B) Chromatogram of recombinant aSyn monomers showing the fractions in which monomeric aSyn was recovered. (PDF 190 kb)

Additional file 2: **Table S1.** Results of the Rab protein screen. Rab-GTPase family members selected in a screen where we assessed alterations in the subcellular distribution of the Rab protein or the colocalization with aSyn in cells treated with aSyn monomers or fibrils. In the column “morphology”, a “11% more Rab-vesicles” statement means that in the 11% of the cells analysed, the localization of Rabs is more vesicular (suggesting an increase of 11% in the active, GTP-bound Rab protein) compared to the localization pattern shown in naïve cells. In contrast, the statement “15% more cytosolic Rab” indicates that, in this case, 15% of the cells analysed showed an increase in the cytosolic, diffuse localization of Rab protein when compared to the naive cells (suggesting an increase of 15% in the inactive, GDP-bound Rab protein). (PDF 396 kb)

Additional file 3: **Figure S2.** Membrane binding and internalization of the A11P/V70P aSyn mutant. (A) Membrane binding properties of WT (left) and of A11P/V70P aSyn (right) in the presence of artificial small unilamellar vesicles membranes (SUVs) [1:100 protein:SUVs ratio]. (B) Immunoblotting of Rab 4A-GFP-expressing cells treated with 1 μM or 5 μM of WT or A11P/V70P aSyn. (C) Quantification of the immunoblots. Dotted bars refer to the band corresponding to aSyn dimers (aSyn**), and clear bars refer to aSyn monomers (aSyn*). Statistical tests were performed using one-way-analysis of variance (ANOVA) with repeated-measures for grouped analysis, followed by Tukey’s post-hoc tests. Data were expressed as mean ± SEM and a 0.5% general significance level was defined, with significance levels as follows: *: *p* < 0.05; **: *p* < 0.01; ***: *p* < 0.001. Significance is shown with the symbol “#” for the monomers, with the symbol “+” for the dimers and with the symbol “*” for the sum between monomers and dimers. (D) ICC of H4 cells transfected with Rab 4A-GFP and treated with 1 μM or 5 μM of aSyn wild type and aSyn A11P/V70P. Scale bar: 30 μm. (PDF 3696 kb)

Additional file 4: **Figure S3.** Dyngo blocks whereas PitStop enhances the internalization of aSyn. (A) ICC of H4 cells treated with 1 μM aSyn monomers and with vehicle, PitStop 30 μM or Dyngo 50 μM. Both PitStop and Dyngo are inhibitors of the endocytic processes. (B) Quantification of the aSyn mean fluorescence intensity in the three conditions. Scale bar: 30 μm. (C) Immunoblotting of H4 cells treated with different concentrations of PitStop and Dyngo. (D) Quantification of the immunoblot. Dotted bars refer to the band corresponding to aSyn dimers (aSyn**), and clear bars refer to aSyn monomers (aSyn*). Statistical tests were performed using one-way-analysis of variance (ANOVA), with repeated-measures for grouped analysis, followed by Tukey’s post-hoc tests. Data were expressed as mean ± SEM and a 0.5% general significance level was defined, with significance levels as follows: *: *p* < 0.05; **: *p* < 0.01; ***: *p* < 0.001. Significance is shown with the symbol “#” for the monomers, with the symbol “+” for the dimers and with the symbol “*” for the sum between monomers and dimers. Scale bar: 30 μm (PDF 1051 kb)

Additional file 5: **Figure S4.** Blocking of autophagy inhibits the degradation of aSyn. (A) ICC of H4 cells treated with 1 μM aSyn monomers and with vehicle, Bafilomycin 100 nM (Baf 100 nM), or with Chloroquine 50 μM (Chlq 50 μM). Bafilomycin and chloroquine are inhibitors of the ALP. (B) Quantification of the aSyn mean fluorescence intensity in the three conditions. Scale bar: 30 μm. (C) Immunoblotting of H4 cells treated with 1 μM aSyn WT and incubated with Bafilomycin 100 nM or Chloroquine 50 μM. (D) quantification of the immunoblot in panel C. Dotted bars refer to the band corresponding to aSyn dimers (aSyn**), and clear bars refer to aSyn monomers (aSyn*). Statistical tests were performed using one-way-analysis of variance (ANOVA), with repeated-measures for grouped analysis, followed by Tukey’s post-hoc tests. Data is expressed as mean ± SEM and a 0.5% general significance level was defined, with significance levels as follows: *: *p* < 0.05; **: *p* < 0.01; ***: *p* < 0.001. Significance is shown with the symbol “#” for the monomers, with the symbol “+” for the dimers and with the symbol “*” for the sum between monomers and dimers. Scale bar: 30 μm. (PDF 1301 kb)
