# Supplementary figures and images for: Membrane binding, internalization, and sorting of alpha-synuclein in the cell
Source: Acta Neuropathol Commun. 2018 Aug 14;6:79. doi: 10.1186/s40478-018-0578-1 (PMC6090819; doi:10.1186/s40478-018-0578-1)

Figure S1

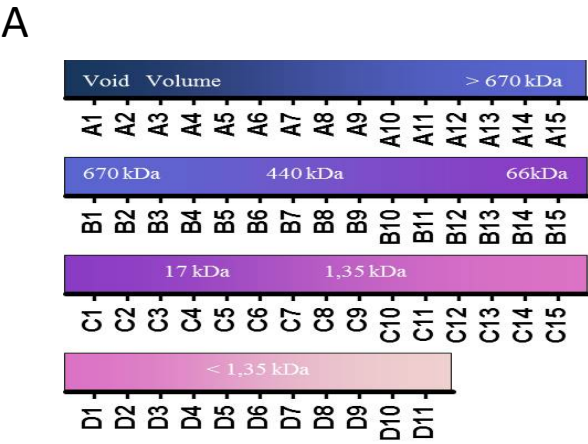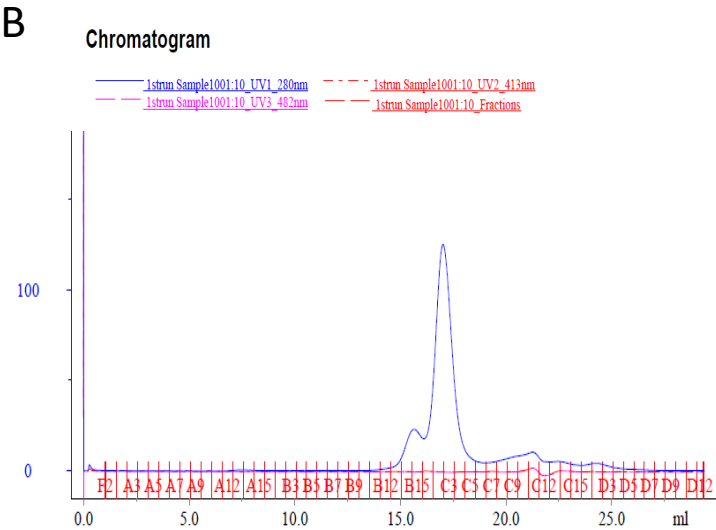

Supplement: Supplementary file 1 — Figure S1. Characterization of recombinant aSyn monomers. (A) Fractions collected upon protein separation on Superose 6 10/300 size exclusion column. (B) Chromatogram of recombinant aSyn monomers showing the fractions in which monomeric aSyn was recovered. (PDF 190 kb) [file 40478_2018_578_MOESM1_ESM.pdf]

A

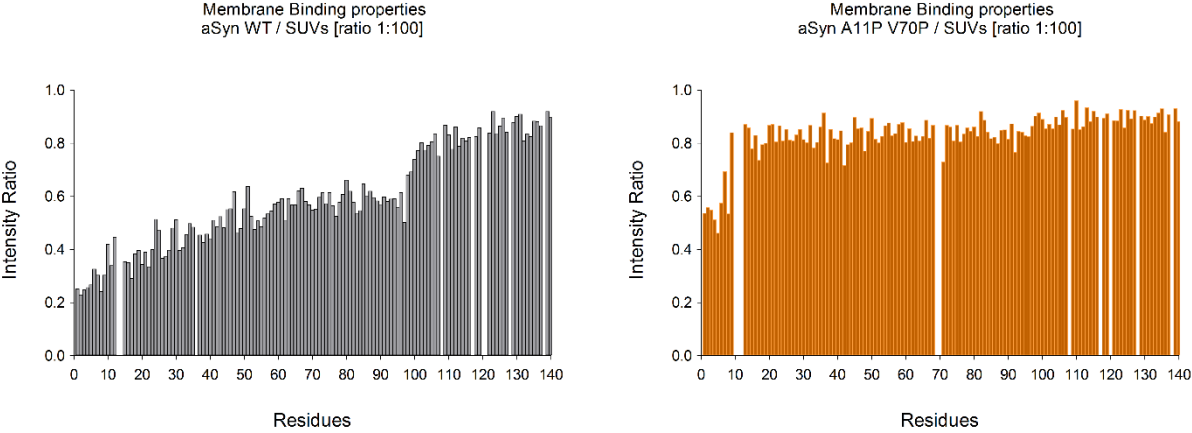

B

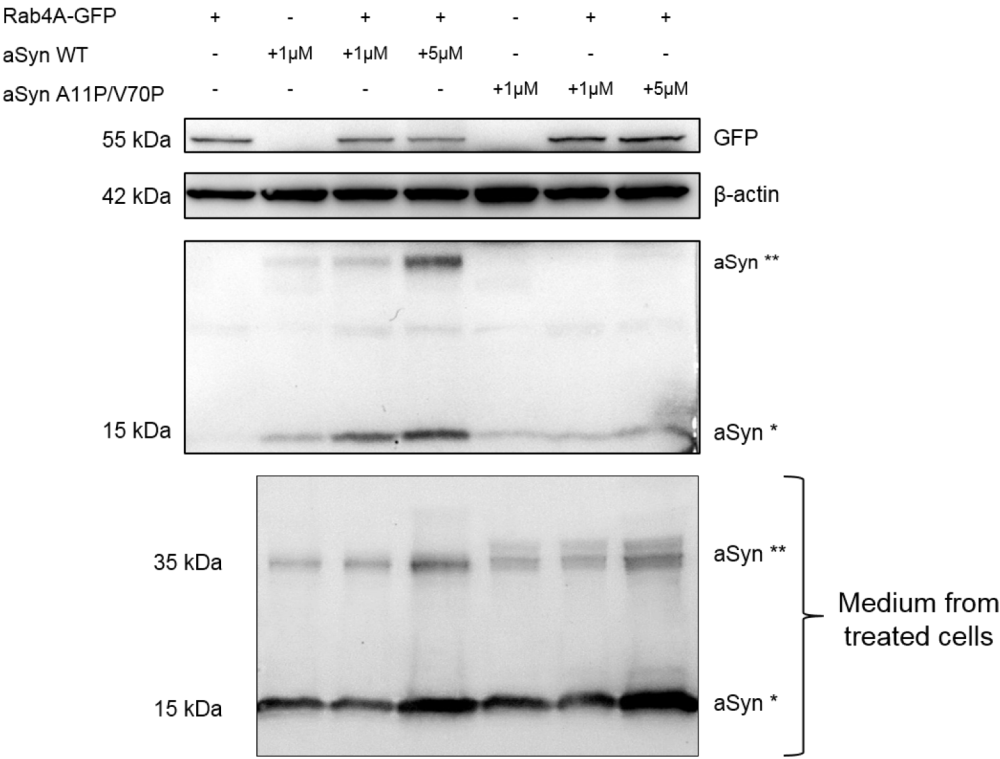

C

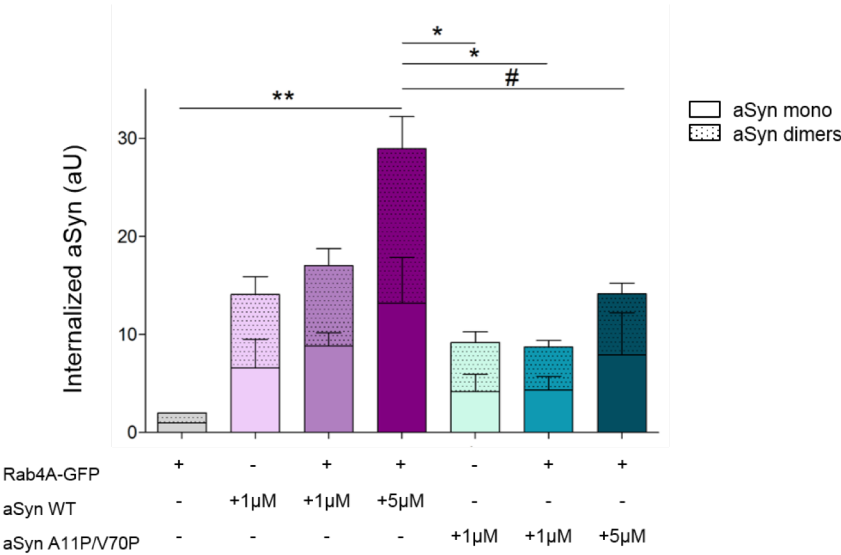

D

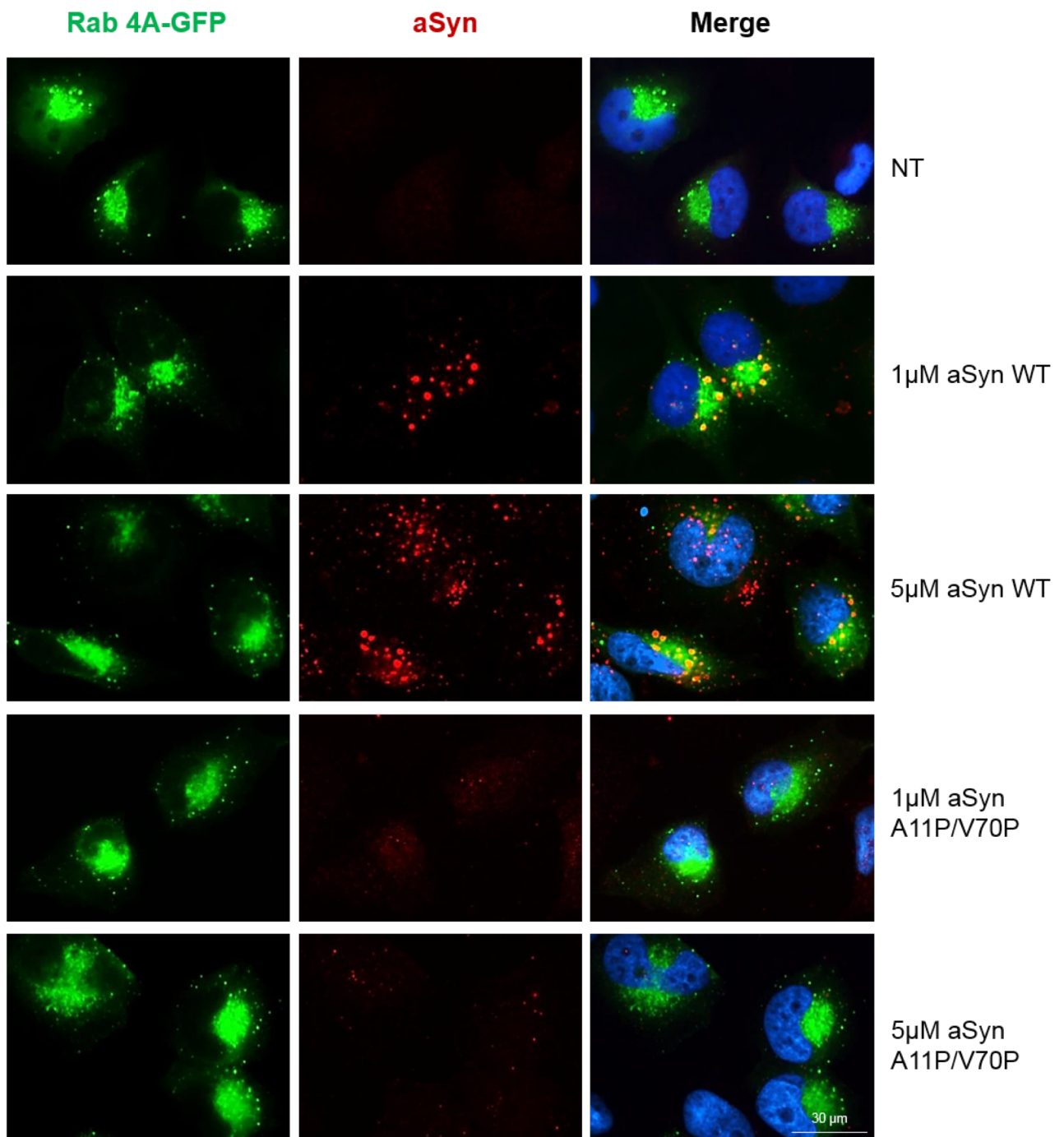

Supplement: Supplementary file 3 — Figure S2. Membrane binding and internalization of the A11P/V70P aSyn mutant. (A) Membrane binding properties of WT (left) and of A11P/V70P aSyn (right) in the presence of artificial small unilamellar vesicles membranes (SUVs) [1:100 protein:SUVs ratio]. (B) Immunoblotting of Rab 4A-GFP-expressing cells treated with 1 μM or 5 μM of WT or A11P/V70P aSyn. (C) Quantification of the immunoblots. Dotted bars refer to the band corresponding to aSyn dimers (aSyn**), and clear bars refer to aSyn monomers (aSyn*). Statistical tests were performed using one-way-analysis of variance (ANOVA) with repeated-measures for grouped analysis, followed by Tukey’s post-hoc tests. Data were expressed as mean ± SEM and a 0.5% general significance level was defined, with significance levels as follows: *: p < 0.05; **: p < 0.01; ***: p < 0.001. Significance is shown with the symbol “#” for the monomers, with the symbol “+” for the dimers and with the symbol “*” for the sum between monomers and dimers. (D) ICC of H4 cells transfected with Rab 4A-GFP and treated with 1 μM or 5 μM of aSyn wild type and aSyn A11P/V70P. Scale bar: 30 μm. (PDF 3696 kb) [file 40478_2018_578_MOESM3_ESM.pdf]

A

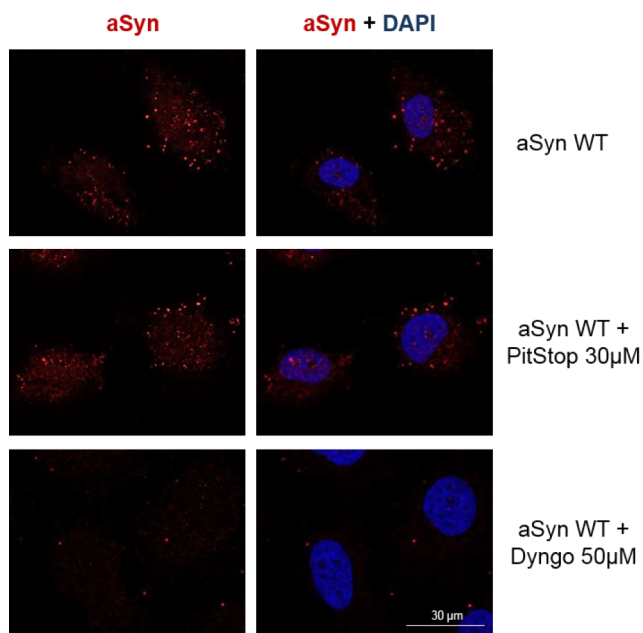

B

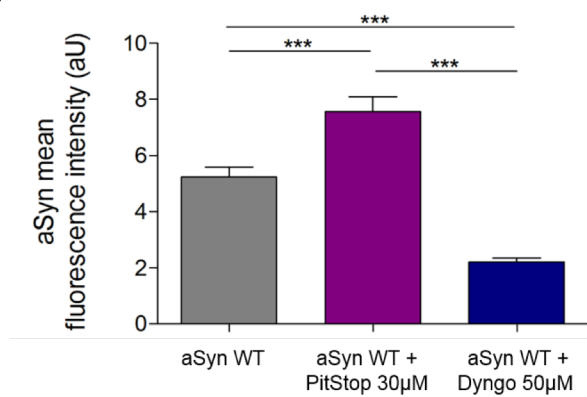

C

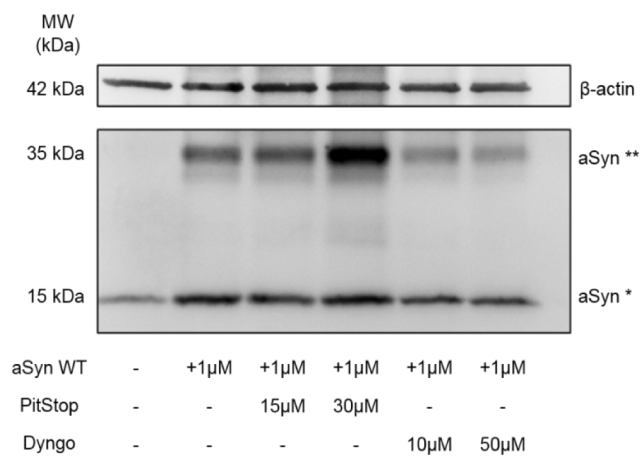

D

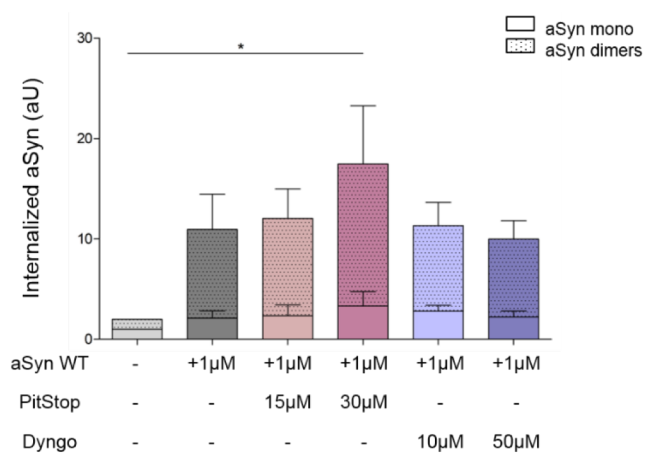

Supplement: Supplementary file 4 — Figure S3. Dyngo blocks whereas PitStop enhances the internalization of aSyn. (A) ICC of H4 cells treated with 1 μM aSyn monomers and with vehicle, PitStop 30 μM or Dyngo 50 μM. Both PitStop and Dyngo are inhibitors of the endocytic processes. (B) Quantification of the aSyn mean fluorescence intensity in the three conditions. Scale bar: 30 μm. (C) Immunoblotting of H4 cells treated with different concentrations of PitStop and Dyngo. (D) Quantification of the immunoblot. Dotted bars refer to the band corresponding to aSyn dimers (aSyn**), and clear bars refer to aSyn monomers (aSyn*). Statistical tests were performed using one-way-analysis of variance (ANOVA), with repeated-measures for grouped analysis, followed by Tukey’s post-hoc tests. Data were expressed as mean ± SEM and a 0.5% general significance level was defined, with significance levels as follows: *: p < 0.05; **: p < 0.01; ***: p < 0.001. Significance is shown with the symbol “#” for the monomers, with the symbol “+” for the dimers and with the symbol “*” for the sum between monomers and dimers. Scale bar: 30 μm (PDF 1051 kb) [file 40478_2018_578_MOESM4_ESM.pdf]

A

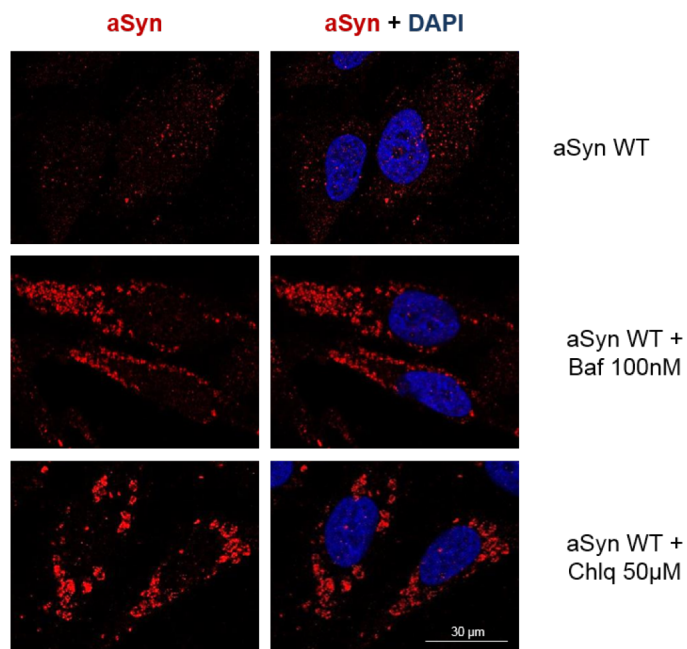

B

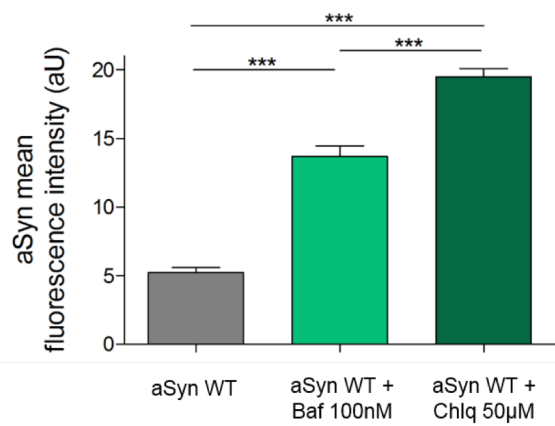

C

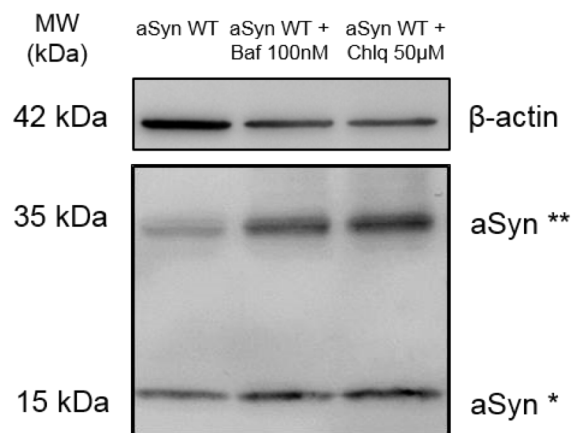

D

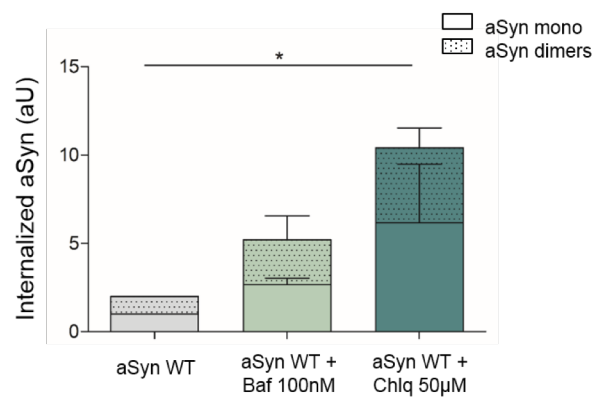

Supplement: Supplementary file 5 — Figure S4. Blocking of autophagy inhibits the degradation of aSyn. (A) ICC of H4 cells treated with 1 μM aSyn monomers and with vehicle, Bafilomycin 100 nM (Baf 100 nM), or with Chloroquine 50 μM (Chlq 50 μM). Bafilomycin and chloroquine are inhibitors of the ALP. (B) Quantification of the aSyn mean fluorescence intensity in the three conditions. Scale bar: 30 μm. (C) Immunoblotting of H4 cells treated with 1 μM aSyn WT and incubated with Bafilomycin 100 nM or Chloroquine 50 μM. (D) quantification of the immunoblot in panel C. Dotted bars refer to the band corresponding to aSyn dimers (aSyn**), and clear bars refer to aSyn monomers (aSyn*). Statistical tests were performed using one-way-analysis of variance (ANOVA), with repeated-measures for grouped analysis, followed by Tukey’s post-hoc tests. Data is expressed as mean ± SEM and a 0.5% general significance level was defined, with significance levels as follows: *: p < 0.05; **: p < 0.01; ***: p < 0.001. Significance is shown with the symbol “#” for the monomers, with the symbol “+” for the dimers and with the symbol “*” for the sum between monomers and dimers. Scale bar: 30 μm. (PDF 1301 kb) [file 40478_2018_578_MOESM5_ESM.pdf]
