# Supplementary material for: Membrane binding, internalization, and sorting of alpha-synuclein in the cell
Source: Acta Neuropathol Commun. 2018 Aug 14;6:79. doi: 10.1186/s40478-018-0578-1 (PMC6090819; doi:10.1186/s40478-018-0578-1)
Supplement: Supplementary file 2 — Table S1. Results of the Rab protein screen. Rab-GTPase family members selected in a screen where we assessed alterations in the subcellular distribution of the Rab protein or the colocalization with aSyn in cells treated with aSyn monomers or fibrils. In the column “morphology”, a “11% more Rab-vesicles” statement means that in the 11% of the cells analysed, the localization of Rabs is more vesicular (suggesting an increase of 11% in the active, GTP-bound Rab protein) compared to the localization pattern shown in naïve cells. In contrast, the statement “15% more cytosolic Rab” indicates that, in this case, 15% of the cells analysed showed an increase in the cytosolic, diffuse localization of Rab protein when compared to the naive cells (suggesting an increase of 15% in the inactive, GDP-bound Rab protein). (PDF 396 kb) [file 40478_2018_578_MOESM2_ESM.pdf]

Table S1

RABs	Localization	Function	H4 cells + aSyn monomers		H4 cells + aSyn fibrils	
			Colocalization	Morphology	Colocalization	Morphology
Rab1A, 1B	ER, Golgi	ER to Golgi, Intra-Golgi	No	11% more RAB-vesicles	No	9% more RAB-vesicles
Rab3A	Secretory vesicles, plasma membrane	Exocytosis, neurotrasmitter release	+	7% more RAB-vesicles	+	15% more RAB-vesicles
Rab4A	Early endosome	Protein recycling/ transport to plasma membrane	+ + +	51% more RAB-vesicles	No	42% more RAB-vesicles
Rab5A	PM, CCVs, early endosome	Early endosome fusion	+ + +	32% more RAB-vesicles	No	35% more RAB-vesicles
Rab6A	Golgi	Endosome to Golgi, intra-Golgi transport Golgi to ER	No	46% more RAB-vesicles	No	41% more RAB-vesicles
Rab7	Late endosome, lysosome, melanosomes, phagosome	Late endosome to lysosome	+ + +	54% more RAB-vesicles	No	50% more RAB-vesicles
Rab9A	Late endosome	Endosome to TGN	+ + +	No changes in the distribution pattern	No	No changes in the distribution pattern
Rab15	Early/sorting endosome, recycling endosome	Sorting endosome/ recycling endosome to plasma membrane	No	40% smaller RAB-vesicles	+	23% smaller RAB-vesicles
Rab25	Recycling endosome	Recycling endosome to plasma membrane	No	40% more RAB-vesicles, 15% more cytosolic RAB	No	18% more RAB-vesicles, 48% more cytosolic RAB
Rab27B	Melanosome	Exocytosis	+	41% more RAB-vesicles	No	25% more RAB-vesicles
Rab33A, 33B	Golgi, dense-core vesiscles	Autophagosome formation	No	18% more RAB-vesicles	No	35% more RAB-vesicles
